# DDMut: predicting effects of mutations on protein stability using deep learning

**DOI:** 10.1093/nar/gkad472

**Published:** 2023-06-07

**Authors:** Yunzhuo Zhou, Qisheng Pan, Douglas E V Pires, Carlos H M Rodrigues, David B Ascher

**Affiliations:** School of Chemistry and Molecular Biosciences, The University of Queensland, Brisbane, Australia; Computational Biology and Clinical Informatics, Baker Heart and Diabetes Institute, Melbourne, Victoria, Australia; School of Chemistry and Molecular Biosciences, The University of Queensland, Brisbane, Australia; Computational Biology and Clinical Informatics, Baker Heart and Diabetes Institute, Melbourne, Victoria, Australia; School of Computing and Information Systems, University of Melbourne, Melbourne, Victoria, Australia; School of Chemistry and Molecular Biosciences, The University of Queensland, Brisbane, Australia; Computational Biology and Clinical Informatics, Baker Heart and Diabetes Institute, Melbourne, Victoria, Australia; School of Chemistry and Molecular Biosciences, The University of Queensland, Brisbane, Australia; Computational Biology and Clinical Informatics, Baker Heart and Diabetes Institute, Melbourne, Victoria, Australia

## Abstract

Understanding the effects of mutations on protein stability is crucial for variant interpretation and prioritisation, protein engineering, and biotechnology. Despite significant efforts, community assessments of predictive tools have highlighted ongoing limitations, including computational time, low predictive power, and biased predictions towards destabilising mutations. To fill this gap, we developed DDMut, a fast and accurate siamese network to predict changes in Gibbs Free Energy upon single and multiple point mutations, leveraging both forward and hypothetical reverse mutations to account for model anti-symmetry. Deep learning models were built by integrating graph-based representations of the localised 3D environment, with convolutional layers and transformer encoders. This combination better captured the distance patterns between atoms by extracting both short-range and long-range interactions. DDMut achieved Pearson's correlations of up to 0.70 (RMSE: 1.37 kcal/mol) on single point mutations, and 0.70 (RMSE: 1.84 kcal/mol) on double/triple mutants, outperforming most available methods across non-redundant blind test sets. Importantly, DDMut was highly scalable and demonstrated anti-symmetric performance on both destabilising and stabilising mutations. We believe DDMut will be a useful platform to better understand the functional consequences of mutations, and guide rational protein engineering. DDMut is freely available as a web server and API at https://biosig.lab.uq.edu.au/ddmut.

## Graphical Abstract

**Graphical Abstract:**
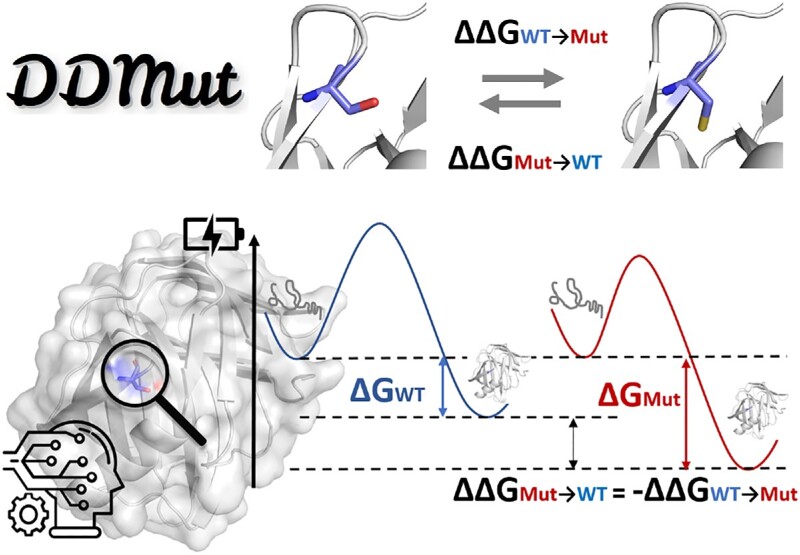

## INTRODUCTION

Proteins are versatile and dynamic tools tailored by nature over the course of evolution to coordinate a range of biochemical processes central to life. They are involved in many biological processes, including cell signalling, proliferation, metabolism, and cell death (1–4). It is, therefore, essential to understand how changes in the protein sequence might impact its structure, function and interactions, giving rise to different phenotypes.

Characterising the molecular consequences of mutations can provide key insights into their biological outcomes. Over the past few years, missense mutations have been extensively studied due to the accumulation of data and their subtle effect on proteins (5). A single amino acid change at the protein sequence level can lead to local atomic changes in the 3D structure, thereby affecting the kinetics of protein folding, stability, flexibility and dynamics (6). Despite large advances in protein structural modelling tools, these changes, however, are currently poorly captured by protein structure prediction tools.

Significant efforts have been invested into understanding and predicting the molecular consequences of mutations in protein coding regions, however most approaches have been limited in their throughput (preventing genome wide and saturation mutagenesis implementation), restricted to predicting consequences of single point missense mutations, and are poorly predictive of stabilising mutations, essential for biotechnological applications, due to inherent biases in the data (7,8).

**Figure 1.**
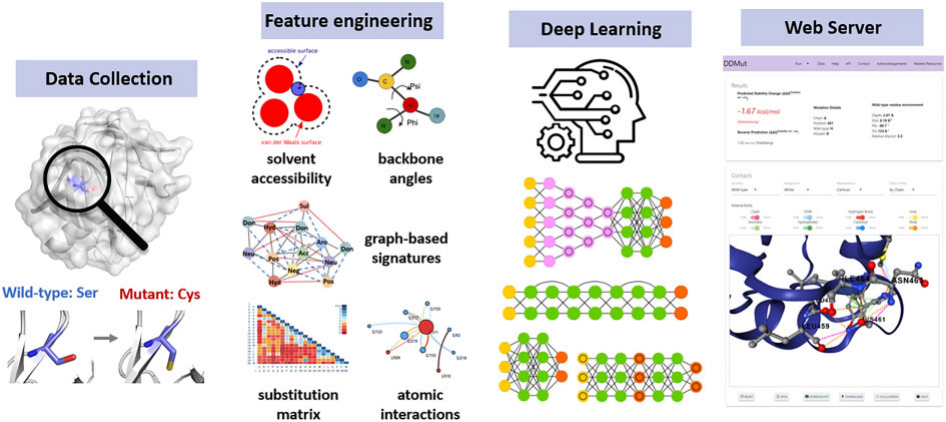
DDMut Workflow. There were four steps involved in the methodology. Firstly, datasets were curated from different sources, and protein structures were curated from RCSB PDB. Secondly, a set of features capturing both geometric and physicochemical properties were generated and normalised. These features were then input into neural networks, which were further optimised via tuning the hyperparameters and layers based on the training performance, and validated on non-redundant blind test sets. Finally, the predictive models were made freely available as easy-to-use web interfaces.

To fill this gap, here we report DDMut, a user-friendly web server that implements our well validated concept of graph-based signatures within a novel deep learning framework (Figure 1), enabling us to rapidly screen both single and multiple point mutations, with comparable performance on both stabilising and destabilising mutations. DDMut was made available as an easy-to-use web server and API, for seamless integration with analytical pipelines at https://biosig.lab.uq.edu.au/ddmut/.

## MATERIALS AND METHODS

### Datasets

#### Training set

DDMut training set for predicting the effects of single point mutations was curated from S2648 (9–12) (originally from ProTherm (13)) and FireProtDB (14). Redundant entries (at mutation level) in the blind test sets were removed from the training set if they have the same Uniprot ID and the same mutation. This is followed by removing the duplicates (at mutation level) in each dataset, where the chosen ΔΔG among the duplicates was measured under physiological conditions closest to the pH of 7 and the temperature of 25°C. To balance the ΔΔG distribution, the hypothetical reverse mutations were introduced into each dataset under the following scheme, where $\Delta G$ is defined as the unfolding free energy:


$$\begin{eqnarray*} \Delta \Delta {G_{Mut \to WT}} &=& \Delta {G_{WT}}\; - \Delta {G_{Mut}}\\ &=& - ({\Delta {G_{Mut}} - \Delta {G_{WT}}})\\ &=& - \Delta \Delta {G_{WT \to Mut}} \end{eqnarray*}$$

This led to our final DDMut training set S9028 (9028 mutations, across 153 proteins). The ΔΔG distribution of S9028 is shown in Supplementary Figure S1.

For predicting the effects of multiple mutations, we removed duplicates and redundant entries (at multiple-mutation level) from DynaMut2 training set (15), and included reverse mutations. This led to our training set SM1242 (98 structures across 94 proteins). We also implemented a protein-level non-redundancy split, which led to the training set SM1218 (67 structures across 60 proteins).

#### Blind test sets

For single point mutations, the five universal non-redundant blind test sets at protein or mutation level for most available protein stability predictors were used for the purpose of benchmark comparisons. S276 (16) and S669 (17) include proteins which have low sequence identity with the original ProTherm (13) dataset and are non-redundant at protein level, whereas S1342 (18) is a blind test non-redundant at mutation level, which means mutations in this dataset may occur on the same protein with mutations in the training set, but at different positions or the same position with different mutant residues. Deep mutational scanning (DMS) datasets from the CAGI5 challenge (19) (including variants for PTEN and TPMT) and Gerasimavicius *et al.* (20), which includes functional scores of 161,441 variants across 45 independent assays, were also evaluated. After removing redundant data within the same test set (at mutation level) and including the hypothetical reverse mutations, our final blind test sets comprised of 552 (37 structures on 37 proteins), 1,304 (94 structures on 87 proteins), 2,024 (129 structures on 120 proteins) mutations. The overlaps between these three datasets at both mutation and protein level are shown in Supplementary Figure S2. Since the CAGI5 challenge data infer protein stability changes from the abundance of EGFP fused to the mutant protein, PTEN (3,736 mutations) and TPNT (3,627 mutations), hypothetical reverse mutations were not included for these datasets. Similarly, reverse mutations were not included for the DMS datasets from Gerasimavicius *et al.* as they indicate the functional impacts of mutations and do not reflect the thermodynamic cycle of protein stability per se.

The blind test set for predicting multiple mutations was originally reported in DynaMut2 (15). Removing the duplicates and including the reverse mutations led to our multiple point mutation blind test set SM420 (61 structures in 63 proteins, 420 double and triple mutations). Under the protein-level non-redundancy split, the blind test set SM444 has 44 structures across 44 proteins.

The wild-type structures for all the datasets were downloaded from Protein Data Bank (21), and the mutant structures were generated from the corresponding wild-type using MODELLER (22) with its default minimisation pipeline. Both wild-type and mutant structures were utilised for generating the features for both forward and reverse mutations.

### Feature engineering

Two sets of features were generated, graph-based signatures and complementary features:

- The graph-based signatures were generated using mCSM (12), a Cut-off Scanning Algorithm (23) operated within a graph-based representation of local residue environment, to capture the distance patterns between pairs of atoms labelled with eight different pharmacophores (Hydrophobics, Positives, Negatives, Hydrogen Acceptors, Hydrogen Donors, Aromatics, Sulphurs and Neutrals)
- The complementary features include both sequence- and structure-based features: (a) sequence-based features were calculated using substitution matrices such as AAindex (24) to capture changes in physico­chemical and biochemical properties, BLOSUM and PAM which are based on sequence alignment; (b) structure-based features include solvent accessibility, residue depth, secondary structure, atomic interactions between the residue of interest and its neighbouring residues calculated by Arpeggio, as well as the changes in interactions upon mutations (25). The tools used to calculate each set of complementary features are detailed in Supplementary Table S1.

The generated features were then normalised by their mean values and standard deviations.

### Network architecture

For both predictive tasks, single and multiple point mutations, DDMut models were trained using siamese networks (Supplementary Figure S3), where two sub-networks with the same architecture and weights were applied on the features calculated from forward and reverse mutations separately. In each sub-network, graph-based signatures were processed with convolutional layers followed by a transformer encoder, whereas complementary features were processed with two dense layers. These two feature components were then concatenated along with residual connections, and then followed by a dense layer. In the end, a contrastive loss function adapted and modified from (26) was calculated to not only consider the errors between the predicted and actual ΔΔ*G*, but also take into account the antisymmetry (errors between forward and the corresponding reverse mutations):


$$\begin{eqnarray*}Loss &=& logcosh\left( {\frac{{\Delta \Delta {G_{Forward}} - \Delta \Delta {G_{Reverse}}}}{2}- y} \right) \\ &&+ \;\left| {\Delta \Delta {G_{Forward}}\; + \;\Delta \Delta {G_{Reverse}}} \right|,\end{eqnarray*}$$

where $\Delta \Delta {G_{Forward}}$ and $\Delta \Delta {G_{Reverse}}$ are predictions for forward and the corresponding reverse mutations, $y$ is the experimental ΔΔG for the forward mutation. For a perfectly anti-symmetric and accurate model, the following rules will be satisfied:


$$\begin{eqnarray*}\Delta \Delta {G_{Forward}} &=& y,\\ \Delta \Delta {G_{Reverse}} &=& - \Delta \Delta {G_{Forward}},\end{eqnarray*}$$

which results in a loss of 0.

During the training phase, the hyperparameters of the architecture were fine-tuned based on the cross-validation performance on the training set. This process was carried out independently for single and multiple point mutation models. The evaluation metrics used include Pearson's (*r*), Kendall's (*k*) and Spearman's (*s*) correlations, root mean square error (RMSE), mean absolute error (MAE) and mean signed error (MSE).

## WEB SERVER

We deployed DDMut as a freely available and user-friendly web server at https://biosig.lab.uq.edu.au/ddmut/. The frontend is built using MaterializeCSS (version 1.0.0), and the backend uses the Flask module (2.0.3) from Python. The web server is hosted on a Linux machine running Nginx.

### Input

DDMut can be used to predict ΔΔGs for both single point mutations and multiple mutations under two different options. Users are required to provide a protein of interest by either uploading a PDB file or input a valid PDB accession code. Mutation details for the ‘Single Mutation’ option (Supplementary Figure S4) can be provided manually as a text string (in the format of wild-type residue one-letter code followed by residue position and mutant residue one-letter code) and Chain identifier, or by uploading a text file with a list of mutations. Alternatively, users can run automated alanine scanning. For the ‘Multiple Mutations’ option (Supplementary Figure S5), mutations should be separated by a semi-colon for each entry (‘A F7A;A V13M’ as an example, where two mutations F7A and V13M are on the same chain A). Here, we are considering double and triple mutations only. Although the web server does accept submissions for more than three simultaneous mutations, it is important to note that the model has only been validated on up to triple point mutations. Users should therefore exercise caution when submitting more than three simultaneous mutations. In both options, users may choose to include predictions for hypothetical reverse mutations, and provide an email address which will be used to send notification once the job's results are ready.

To assist users with job submission, a help page is available at https://biosig.lab.uq.edu.au/ddmut/help.

### Output

For ‘Single Mutation’, predicted ΔΔG values are displayed alongside information of the wild-type residue environment and a 3D interactive viewer built using NGL viewer (27), highlighting inter-residue interactions (Supplementary Figure S6). For ‘Mutation List’, results are summarised as a downloadable table with buttons redirecting to the detail page, which is similar to the ‘Single Mutation’ outputs of each variation (Supplementary Figure S7). For ‘Alanine Scanning’, predictions are mapped onto the protein sequence and 3D structure displayed using NGL viewer, and results are downloadable either in the format of a table, or a 3D protein structure with the predictions annotated on the *b*-factor column (Supplementary Figure S8).

For ‘Multiple Mutations’, results are summarised as a downloadable table, and users can select specific entries from the table to be highlighted in the interactive viewer with residue contacts (Supplementary Figure S9).

### API

DDMut provides an API (Application Programming Interface) to facilitate convenient integration into different research pipelines. A unique ID will be assigned to each single submitted job, and can be used to query the job status or access the website interface. Our API requires the same inputs as our website. More detailed explanations and examples using curl and Python can be found at https://biosig.lab.uq.edu.au/ddmut/api.

### Processing time

The processing time of DDMut was compared with DynaMut (28), DynaMut2 (15), MAESTRO (29), FoldX (30), DDGun (31) and BoostDDG (32) (Supplementary Table S2) using proteins with different sequence lengths. DDMut demonstrated competitive efficiency especially for larger proteins (Supplementary Figure S10).

## VALIDATION

DDMut was able to accurately and robustly predict the effects of both single and multiple point mutations. The performance on the training sets (under 10-fold cross-validation) and blind test sets are shown in Table 1.

**Table 1.** DDMut performance on training set (under 10-fold cross-validation) and blind-test sets

		Pearson (*r*)	Spearman (s)	Kendall (*k*)	RMSE (kcal/mol)	MAE (kcal/mol)	MSE (kcal/mol)
Single mutation	S9028 cross-val	0.70	0.67	0.49	1.37	1.00	−0.002
	S552	0.60	0.54	0.39	1.14	0.86	−0.02
	S1304	0.62	0.61	0.45	1.50	1.08	−0.03
	S2024	0.40	0.41	0.29	2.24	1.54	−0.06
	CAGI5	0.48	0.42	0.29	−*	−*	−*
Multiple mutation	SM1242 cross-val	0.69	0.74	0.56	1.83	1.27	0.02
	SM420	0.70	0.73	0.54	1.84	1.30	−0.003
	SM1218 cross-val	0.45	0.45	0.31	2.17	1.69	0.04
	SM444	0.49	0.55	0.39	2.45	1.84	0.07

*Since the labels of the CAGI5 dataset indicate the abundance of EGFP instead of ΔΔG, RMSE, MAE and MSE were not measured.

### Predicting the effects of single point mutations

**Figure 2.**
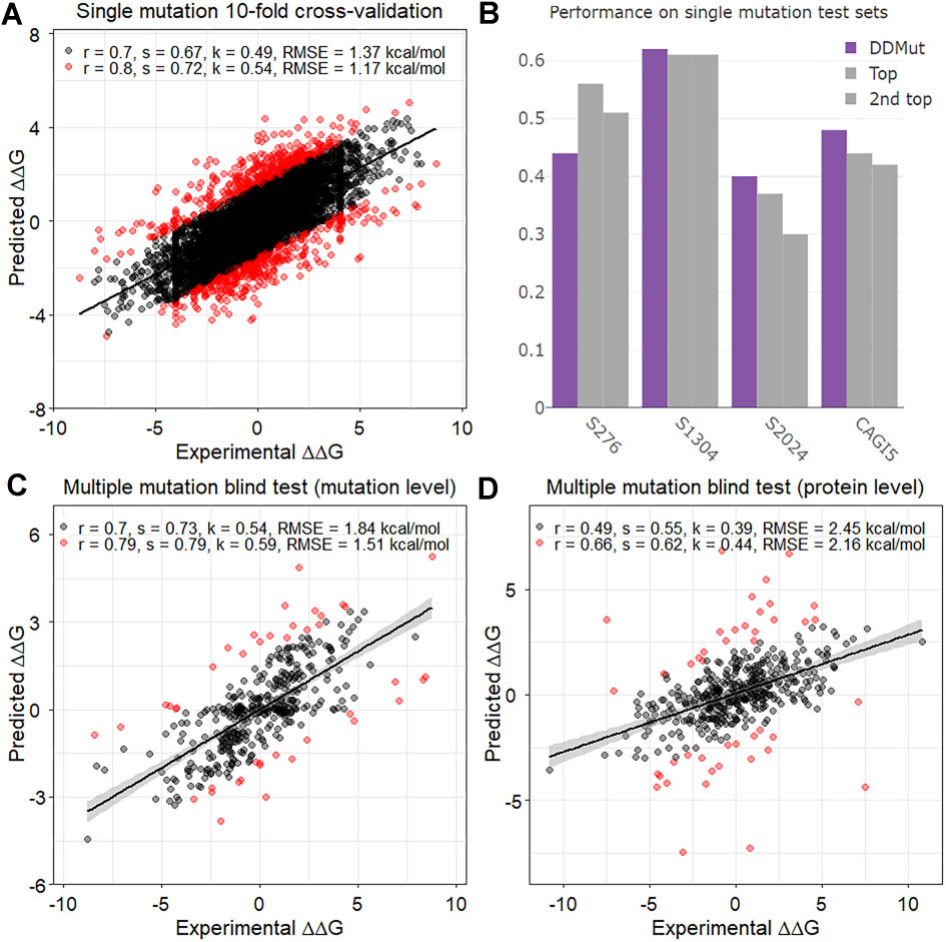
DDMut performance on the training and blind test sets. (**A**) Cross-validation performance on the single mutation training set, where mutations on the same protein were all split into the same fold to reduce redundancy. The 10% outliers are coloured in red. *r*, *k* and *s* denote Pearson's, Kendall's and Spearman's correlations. (**B**) Performance comparison with the top two methods among all the benchmarking methods. S276 and CAGI5 include forward mutations only, S1304 and S2024 include both forward and the hypothetical reverse mutations. The full benchmarking results are shown in Supplementary Tables S3–S9. (**C**) Performance on the multiple mutation blind test set SM420 (non-redundant at multiple-mutation level). (**D**) Performance on the multiple mutation blind test set SM444 (non-redundant at protein level).

We evaluated the performance of DDMut on our training set comprising 9,028 single point mutations under 10-fold cross-validations. Two different fold-splitting strategies were implemented, low redundancy at the amino acid level and low redundancy at the protein level. Our method achieved a Pearson's correlation of 0.77 (RMSE: 1.25 kcal/mol) under the amino acid low redundancy scheme, and 0.70 (RMSE: 1.37 kcal/mol) for the protein low redundancy split (Figure 2A). The comparable performance between the two splits provided confidence in the robustness of the models. DDMut also achieved consistent performance across both forward and reverse mutations (RMSE: 1.36 and 1.38 kcal/mol respectively), with a Pearson's correlation of –0.93 between the forward and corresponding reverse mutations, indicating high model anti-symmetry (Supplementary Figure S11). To build a more robust model capable of predicting mutation effects on stability changes of a broader group of proteins, the hyperparameters in the neural network were tuned based on the cross-validation performance under the protein low redundancy split. The final model was trained on the entire training set, and then evaluated on blind test sets.

To fairly compare our model with other available methods, we tested DDMut on non-redundant blind test sets comprising 276, 1,304 and 2,024 mutations, and a DMS dataset from the CAGI5 challenge including variants for PTEN and TPMT. Our model achieved the top performance for three out of the four blind test sets (Figure 2B), with consistent performance on both forward and the hypothetical reverse mutations, and on both stabilising (ΔΔ*G* ≥ 0 kcal/mol) and destabilising (ΔΔ*G* < 0 kcal/mol) mutations (Figure 2B, Supplementary Tables S3–S9). This provided confidence in the generalisability of the DDMut model. We then further evaluated the capability of DDMut on predicting the functional scores of 161,441 variants across 45 independent DMS assays, ranging from protein abundance, protein binding, activity assays, growth experiments and viral replication (20). DDMut demonstrated competitive performance when compared to nine other protein stability predictors, while the performance is highly heterogeneous across different assay types (Supplementary Figure S12).

The contribution of each architecture component was evaluated using ablation studies. By disabling sub-blocks in the network architecture, we found all the components were essential for the final predictions. While the two dense layers contributed more to the final performance, the transformer encoder and convolutional layers contributed less (Supplementary Table S10). This can also be caused by the sets of features they processed respectively. To further understand what makes a mutation to be stabilising (ΔΔ*G* ≥ 0 kcal/mol) or destabilising (ΔΔ*G* < 0 kcal/mol), we then evaluated the importance of each feature to the neural network by a model-agnostic approach, i.e. feature permutation importance. We randomly shuffled the feature values across all the mutations in the balanced blind test set S1304, while maintaining the mean and variance of the feature. Notably, the top two important features (Δ hydrophobic contacts; Δ hydrophobic atoms) are both related to the changes in hydrophobicity upon mutations, shuffling each of them alone dropped the Pearson's correlation on forward mutations by around 0.05 (Supplementary Table S11).

### Predicting the effects of multiple point mutations

We then evaluated the performance of DDMut on predicting the effects of double/triple point mutations. To keep consistency with data used by other methods, DDMut was trained and optimised on a dataset consisting of 1242 entries (SM1242), and performance was assessed using a blind test non-redundant at multiple-mutation level comprising 420 entries (SM420). On the training set SM1242, DDMut achieved Pearson's correlations of 0.69 (RMSE: 1.83 kcal/mol) under 10-fold cross validation using the amino acid low redundancy splitting scheme. On the blind test SM420, DDMut achieved a Pearson's correlation of 0.70 (RMSE: 1.84 kcal/mol) (Figure 2C), outperforming previous methods on the forward mutations with consistent performance on both stabilising and destabilising mutations (Supplementary Table S12), and also on both double and triple point mutations (Supplementary Table S13).

Under the protein-level non-redundancy scheme, we trained and optimised DDMut on SM1218, and tested on SM444. On the training set SM1218, DDMut achieved a Pearson's correlation of 0.45 (RMSE: 2.17 kcal/mol) under 10-fold cross validation. On the blind test SM444 non-redundant at protein level, DDMut achieved a Pearson's correlation of 0.49 (RMSE: 2.45 kcal/mol) (Figure 2D), comparable to performance across training. The performance on stabilising and destabilising mutations, and on double and triple point mutations are shown in Supplementary Tables S14 and S15, demonstrating that despite a drop in performance, DDMut outperforms previous methods including MAESTRO, FoldX, and DDGun. Due to the limited sample size for multiple mutations and a demand for scalability to unseen proteins, the final model deployed on our web server was built on the combined training and blind test sets.

## CONCLUSION

Here we present DDMut, a fast and accurate tool to investigate the effects of single and multiple missense mutations on protein stability. DDMut is a siamese network utilising both forward and the hypothetical reverse mutations to account for model anti-symmetry, and integrating our well-established graph-based signatures with convolutional layers and transformer encoder to better capture short- and long-range atomic interactions step-wisely within a localised 3D residue environment. DDMut achieved consistent performance on both stabilising and destabilising mutations, and outperformed other tools on different blind-test sets in terms of both accuracy and efficiency. By allowing users to perform alanine scanning, DDMut could also be potentially used for probing important residue side chains for protein folding and stability. We believe DDMut will be an invaluable tool for various applications such as detecting functional residues, inferring disease-associated missense mutations, and engineering more stable proteins. DDMut is freely available at https://biosig.lab.uq.edu.au/ddmut/.

## DATA AVAILABILITY

DDMut web-server and all the datasets used in this study are freely available at https://biosig.lab.uq.edu.au/ddmut/.

## Supplementary Material

gkad472_Supplemental_File
