# Supplementary material for: DDMut: predicting effects of mutations on protein stability using deep learning
Source: Nucleic Acids Res. 2023 Jun 7;51(W1):W122–8. doi: 10.1093/nar/gkad472 (PMC10320186; doi:10.1093/nar/gkad472)
Supplement: gkad472_Supplemental_File [file gkad472_supplemental_file.docx]

**SUPPLEMENTARY MATERIAL**

# **DDMut: Predicting Effects of Mutations on Protein Stability Using Deep Learning**

Yunzhuo Zhou^1,2^, Qisheng Pan^1,2^, Douglas E.V. Pires^3^, Carlos H.M. Rodrigues^1,2^, David B. Ascher^1,2,*^

^1^School of Chemistry and Molecular Biosciences, The University of Queensland, Brisbane, Australia

^2^Computational Biology and Clinical Informatics, Baker Heart and Diabetes Institute, Melbourne, Victoria, Australia

^3^School of Computing and Information Systems, University of Melbourne, Melbourne, Victoria, Australia

*To whom correspondence should be addressed D.B.A. Tel: +61 90354794; Email: d.ascher@uq.edu.au

**Table S1. Complementary features and the tools used for generation.**

| **Based on** | **Feature description** | **Tools** |
| --- | --- | --- |
| Sequence | Amino acid indices/mutation matrices capturing physicochemical and biochemical properties, as well as similarities between amino acids | AAindex (1) |
|  | BLOSUM & PAM substitution matrices calculated based on sequence alignment | Biopython (2) |
|  | Δ pharmacophores | In-house script |
| Structure | Wild-type residue environment: residue depth, residue solvent accessibility, secondary structure, Phi and Psi angles | Biopython (2) |
|  | Atomic interactions within wild-type local residue environment, and the changes in interactions upon mutations | Arpeggio (3) |

**Table S2. Processing time of DDMut, DynaMut1 and DynaMut2 using protein inputs with different sequence lengths. Each tool was run for 10 repetitions.**

|  | 1A43_A | 1AKY_A | 1AMQ_A | 1AON_A | 2ZT8_A | 6M71_A |
| --- | --- | --- | --- | --- | --- | --- |
|  | G156A | V8I | C270A | T516V | T661V | Y884K |
|  | 72 aa | 218 aa | 396 aa | 524 aa | 635 aa | 859 aa |
| **DDMut** | **11.86 ± 0.06** | **17.81 ± 1.88** | **23.45 ± 1.98** | **31.40 ± 0.08** | **41.55 ± 0.09** | **73.30 ± 3.09** |
| DynaMut | 67.50 ± 1.08 | 212.90 ± 2.47 | 373.60 ± 4.33 | 466.30 ± 8.50 | 617.30 ± 4.03 | 1460.50 ± 9.71 |
| DynaMut2 | 16.70 ± 1.89 | 22.00 ± 0.67 | 43.25 ± 0.63 | 81.40 ± 4.62 | 112.10 ± 2.07 | 231.90 ± 5.53 |
| **MAESTRO** | **0.26 ± 0.00** | **0.39 ± 0.01** | **0.61 ± 0.01** | **0.82 ± 0.01** | **0.88 ± 0.00** | **2.62 ± 0.02** |
| **FoldX** | **3.26 ± 0.07** | **3.02 ± 0.03** | **1.56 ± 0.02** | **32.65 ± 0.15** | **3.38 ± 0.02** | **45.99 ± 0.11** |
| DDGun | 71.95 ± 3.67 | 267.09 ± 1.62 | 359.43 ± 9.92 | 403.70 ± 20.71 | 410.26 ± 13.60 | 587.89 ± 67.92 |
| BoostDDG | 128.06 ± 0.07 | 365.02 ± 0.02 | 357.47 ± 0.04 | 385.90 ± 0.04 | 325.36 ± 0.00 | 270.95 ± 0.05 |

**Table S3. Performance on S552 forward mutations (S276).**

|  | Forward | |
| --- | --- | --- |
| Methods | Pearson | MAE |
| **DDMut** | 0.44 | 0.86 |
| DeepDDG | **0.55** | 0.86 |
| DynaMut2 | 0.52 | 0.88 |
| SDM | 0.48 | 1.02 |
| mCSM | 0.47 | 0.90 |
| I-Mutant | 0.45 | 0.91 |
| I-Mutant-Seq | 0.39 | 1.08 |
| STRUM | 0.45 | 0.88 |
| MUpro | 0.19 | 1.06 |
| Rosetta | 0.34 | 5.25 |
| FoldX | 0.30 | 2.13 |
| DFIRE | 0.23 | 1.25 |
| PopMusic | 0.44 | 0.91 |
| EASE-MM | 0.40 | 0.91 |
| INPS | 0.47 | 0.89 |
| BoostDDG-single | 0.36 | 1.02 |
| BoostDDG | 0.51 | **0.78** |

**Table S4. Performance on S1304.**

|  | Overall | | | |
| --- | --- | --- | --- | --- |
| Methods | Pearson | RMSE | MAE | MSE |
| **DDMut** | **0.62** | **1.50** | 1.08 | -0.03 |
| MAESTRO | 0.44 | 1.81 | 1.37 | -0.58 |
| FoldX | 0.30 | 2.40 | 1.54 | -0.31 |
| PremPS | 0.61 | 1.51 | 1.09 | 0.09 |
| Dynamut | 0.50 | 1.66 | 1.22 | -0.06 |
| mCSM | 0.36 | 1.97 | 1.51 | -0.85 |
| SDM | 0.32 | 1.94 | 1.46 | -0.41 |
| DUET | 0.41 | 1.87 | 1.40 | -0.68 |
| I-Mutant3.0 | 0.32 | 1.98 | 1.51 | -0.81 |
| I-Mutant3.0-Seq | 0.36 | 1.93 | 1.48 | -0.76 |
| MuPro | 0.32 | 2.04 | 1.60 | -0.95 |
| SAAFEC-Seq | 0.26 | 2.03 | 1.55 | -0.83 |
| DDGun3D | 0.56 | 1.62 | 1.14 | -0.05 |
| DDGun | 0.56 | 1.78 | 1.28 | -0.06 |
| ACDC-NN-Seq | 0.59 | 1.54 | 1.09 | -4.36 |
| ACDC-NN | 0.61 | 1.51 | **1.06** | -0.02 |
| INPS-Seq | 0.60 | 1.54 | 1.11 | **0** |
| INPS3D | 0.54 | 1.66 | 1.20 | -0.38 |
| PopMusic | 0.45 | 1.84 | 1.38 | -0.69 |
| SOL_ACC | 0.01 | 1.95 | 1.50 | 0.29 |
| ThermoNet | 0.50 | 1.65 | 1.22 | -0.05 |

**Table S5. Performance on S1304 forward and reverse mutations.**

|  | Forward | | | | Reverse | | | |
| --- | --- | --- | --- | --- | --- | --- | --- | --- |
| Methods | Pearson | RMSE | MAE | MSE | Pearson | RMSE | MAE | MSE |
| **DDMut** | 0.45 | 1.50 | 1.07 | 0.24 | **0.44** | 1.51 | 1.09 | -0.31 |
| MAESTRO | **0.49** | **1.46** | 1.07 | 0.12 | 0.20 | 2.11 | 1.66 | -1.27 |
| FoldX | 0.22 | 2.31 | 1.57 | 0.15 | 0.22 | 2.49 | 1.51 | -0.78 |
| PremPS | 0.40 | 1.53 | 1.11 | 0.17 | 0.41 | **1.50** | **1.07** | **0.005** |
| Dynamut | 0.41 | 1.61 | 1.19 | 0.53 | 0.34 | 1.71 | 1.25 | -0.65 |
| mCSM | 0.35 | 1.56 | 1.14 | -0.07 | 0.22 | 2.32 | 1.88 | -1.64 |
| SDM | 0.41 | 1.68 | 1.27 | 0.38 | 0.14 | 2.17 | 1.65 | -1.20 |
| DUET | 0.41 | 1.53 | 1.10 | 0.03 | 0.23 | 2.16 | 1.70 | -1.38 |
| I-Mutant3.0 | 0.35 | 1.55 | 1.14 | **0.03** | 0.15 | 2.33 | 1.88 | -1.66 |
| I-Mutant3.0-Seq | 0.33 | 1.57 | 1.17 | **0.03** | 0.22 | 2.23 | -1.56 | 1.80 |
| MuPro | 0.24 | 1.63 | 1.22 | -0.15 | 0.20 | 2.39 | 1.97 | -1.75 |
| SAAFEC-Seq | 0.35 | 1.55 | 1.14 | 0.03 | 0 | 2.41 | 1.96 | -1.71 |
| DDGun3D | 0.43 | 1.61 | 1.12 | 0.19 | 0.40 | 1.63 | 1.16 | -0.28 |
| DDGun | 0.40 | 1.76 | 1.28 | -0.13 | 0.38 | 1.79 | 1.29 | 0.02 |
| ACDC-NN-Seq | 0.42 | 1.54 | 1.09 | 0.25 | 0.42 | 1.54 | 1.09 | -0.25 |
| ACDC-NN | 0.45 | 1.50 | **1.06** | 0.29 | **0.44** | 1.52 | **1.07** | -0.32 |
| INPS-Seq | 0.42 | 1.54 | 1.11 | 0.07 | 0.42 | 1.54 | 1.12 | -0.07 |
| INPS3D | 0.42 | 1.52 | 1.08 | -0.06 | 0.33 | 1.78 | 1.32 | -0.70 |
| PopMusic | 0.41 | 1.53 | 1.10 | -0.05 | 0.24 | 2.10 | 1.66 | -1.34 |
| SOL_ACC | 0.19 | 2.05 | 1.62 | 1.26 | -0.16 | 1.83 | 1.37 | -0.67 |
| ThermoNet | 0.38 | 1.63 | 1.19 | 0.57 | 0.37 | 1.67 | 1.25 | -0.67 |

**Table S6. Performance on S1304 stabilising and destabilising mutations.**

|  | Stabilising | | | | Destabilising | | | |
| --- | --- | --- | --- | --- | --- | --- | --- | --- |
| Methods | Pearson | RMSE | MAE | MSE | Pearson | RMSE | MAE | MSE |
| **DDMut** | **0.42** | 1.52 | 1.10 | -0.94 | **0.44** | 1.49 | 1.08 | 0.87 |
| MAESTRO | 0.16 | 2.18 | 1.75 | -1.70 | 0.43 | 1.36 | 0.99 | 0.55 |
| FoldX | 0.16 | 2.68 | 1.62 | -1.25 | 0.24 | 2.09 | 1.46 | 0.62 |
| PremPS | 0.39 | **1.50** | **1.06** | -0.74 | 0.43 | 1.53 | 1.12 | 0.92 |
| Dynamut | 0.29 | 1.71 | 1.25 | -1.11 | 0.35 | 1.61 | 1.20 | 1.00 |
| mCSM | 0.11 | 2.48 | 2.10 | -2.09 | 0.36 | 1.28 | 0.92 | 0.38 |
| SDM | 0.03 | 2.21 | 1.69 | -1.55 | 0.34 | 1.63 | 1.24 | 0.73 |
| DUET | 0.11 | 2.30 | 1.85 | -1.82 | 0.40 | 1.33 | 0.96 | 0.47 |
| I-Mutant3.0 | 0.04 | 2.50 | 2.10 | -2.10 | 0.36 | 1.29 | 0.92 | 0.47 |
| I-Mutant3.0-Seq | 0.15 | 2.38 | 2.01 | -2.00 | 0.31 | 1.34 | 0.96 | 0.48 |
| MuPro | 0.11 | 2.57 | 2.22 | -2.22 | 0.20 | 1.33 | 0.97 | **0.32** |
| SAAFEC-Seq | -0.06 | 2.55 | 2.16 | -2.16 | 0.33 | 1.31 | **0.49** | 0.94 |
| DDGun3D | 0.35 | 1.65 | 1.17 | -0.81 | 0.37 | 1.60 | 1.11 | 0.71 |
| DDGun | 0.32 | 1.79 | 1.29 | -0.60 | 0.33 | 1.76 | 1.28 | 0.49 |
| ACDC-NN-Seq | 0.37 | 1.55 | 1.10 | -0.84 | 0.37 | 1.55 | 1.10 | 0.84 |
| ACDC-NN | 0.39 | 1.53 | 1.08 | -0.89 | 0.41 | 1.50 | 1.06 | 0.86 |
| INPS-Seq | 0.37 | 1.54 | 1.12 | **-0.70** | 0.36 | 1.54 | 1.11 | 0.70 |
| INPS3D | 0.24 | 1.92 | 1.46 | -1.26 | 0.41 | 1.34 | 0.95 | 0.50 |
| PopMusic | 0.13 | 2.26 | 1.83 | -1.81 | 0.40 | **1.30** | 0.93 | 0.42 |
| SOL_ACC | -0.22 | 1.76 | 1.26 | -1.15 | 0.27 | 2.12 | 1.74 | 1.74 |
| ThermoNet | 0.34 | 1.68 | 1.26 | -1.15 | 0.33 | 1.64 | 1.19 | 1.05 |

**Table S7. Performance on S2024.**

|  | Overall | | | |
| --- | --- | --- | --- | --- |
| Methods | Pearson | RMSE | MAE | MSE |
| **DDMut** | **0.40** | **2.24** | **1.54** | **-0.06** |
| I-Mutant2.0 | 0.08 | 2.72 | 1.97 | -0.94 |
| FoldX | 0.37 | 3.05 | 1.88 | -0.29 |
| DDGun | 0.30 | 2.41 | 1.67 | -0.04 |
| DUET | 0.17 | 2.58 | 1.84 | -0.80 |
| mCSM | 0.13 | 2.63 | 1.91 | -0.96 |
| SDM | 0.14 | 2.61 | 1.92 | -0.46 |
| ENCOM | 0.03 | 2.75 | 1.77 | 0.10 |
| DynaMut1 | 0.23 | 2.40 | 1.67 | -0.02 |
| SAAFEC | 0.13 | 2.60 | 1.88 | -0.96 |
| DynaMut2 | 0.11 | 2.62 | 1.87 | -0.80 |
| MAESTRO | 0.20 | 2.52 | 1.80 | -0.64 |

**Table S8. Performance on S2024 forward and reverse mutations.**

|  | Forward | | | | Reverse | | | |
| --- | --- | --- | --- | --- | --- | --- | --- | --- |
| Methods | Pearson | RMSE | MAE | MSE | Pearson | RMSE | MAE | MSE |
| **DDMut** | 0.27 | **2.22** | **1.54** | **-0.01** | 0.23 | **2.25** | **1.55** | -0.10 |
| I-Mutant2.0 | 0.13 | 2.42 | 1.68 | -0.19 | -0.03 | 3.00 | 2.27 | -1.69 |
| FoldX | **0.30** | 3.01 | 1.87 | -0.52 | **0.27** | 3.10 | 1.89 | **-0.06** |
| DDGun | 0.17 | 2.41 | 1.67 | 0.17 | 0.16 | 2.41 | 1.68 | -0.25 |
| DUET | 0.18 | 2.32 | 1.59 | -0.13 | 0.04 | 2.82 | 2.10 | -1.46 |
| mCSM | 0.15 | 2.32 | 1.61 | -0.26 | 0 | 2.91 | 2.20 | -1.65 |
| SDM | 0.17 | 2.46 | 1.77 | 0.35 | 0.12 | 2.76 | 2.06 | -1.28 |
| ENCOM | 0.03 | 3.03 | 1.87 | 0.88 | 0 | 2.43 | 1.68 | -0.68 |
| DynaMut1 | 0.19 | 2.34 | 1.64 | 0.48 | 0.05 | 2.45 | 1.71 | -0.52 |
| SAAFEC | 0.20 | 2.25 | 1.55 | -0.21 | -0.06 | 2.91 | 2.21 | -1.71 |
| DynaMut2 | 0.09 | 2.40 | 1.65 | -0.13 | 0.01 | 2.83 | 2.09 | -1.47 |
| MAESTRO | 0.25 | 2.26 | 1.57 | -0.03 | 0 | 2.76 | 2.03 | -1.26 |

**Table S9. Performance on CAGI5 dataset.**

| Methods | PTEN | TPMT | TPMT & PTEN |
| --- | --- | --- | --- |
| **DDMut** | **0.49** | **0.47** | **0.48** |
| MUpro1.1 | 0.22 | 0.24 | 0.23 |
| I-Mutant2.0 | 0.20 | 0.25 | 0.23 |
| EASE-MM | 0.41 | 0.38 | 0.39 |
| STRUM | 0.13 | 0.43 | 0.32 |
| INPS | 0.44 | 0.38 | 0.42 |
| BoostDDG | 0.46 | 0.42 | 0.44 |

**Table S10. Ablation study tested on S1304. The disabled blocks refer to the DDMut architecture displayed in Figure S2B.**

| Disabled block | Overall | | Forward | |
| --- | --- | --- | --- | --- |
|  | Pearson | RMSE | Pearson | RMSE |
| No disabling | 0.62 | 1.50 | 0.45 | 1.50 |
| others1D + Dense1 & 2 | 0.51 | 1.67 | 0.21 | 1.66 |
| gbs2D + Conv + TransformerEncoder | 0.58 | 1.56 | 0.39 | 1.56 |
| Transformer Encoder | 0.58 | 1.56 | 0.40 | 1.56 |
| Convolution | 0.56 | 1.59 | 0.41 | 1.58 |

**Table S11. Feature permutation importance tested on S1304.**

|  | | Overall | | Forward | |
| --- | --- | --- | --- | --- | --- |
| Shuffled feature(s) | | Pearson | RMSE | Pearson | RMSE |
| No shuffling | | 0.62 | 1.50 | 0.45 | 1.50 |
| Single feature | Δ hydrophobic contacts | 0.59 | 1.54 | 0.41 | 1.53 |
|  | Δ hydrophobic atoms | 0.57 | 1.57 | 0.37 | 1.57 |
| Group of features | graph-based signatures | 0.57 | 1.57 | 0.37 | 1.56 |
|  | mutant structure based | 0.57 | 1.57 | 0.42 | 1.53 |
|  | wildtype structure based | 0.55 | 1.59 | 0.29 | 1.63 |
|  | Amino acid change | 0.5 | 1.66 | 0.28 | 1.65 |

**Table S12. Performance comparison on multiple mutation blind test SM420 (non-redundant at multiple mutation level) forward mutations split by stabilising and destabilising mutations.**

|  | Overall | | Stabilising | | Destabilising | |
| --- | --- | --- | --- | --- | --- | --- |
| Methods | Pearson | RMSE | Pearson | RMSE | Pearson | RMSE |
| **DDMut** | **0.66** | **1.83** | **0.29** | **1.80** | **0.52** | **1.86** |
| MAESTRO | 0.19 | 2.50 | 0.10 | 1.91 | 0.24 | 2.81 |
| FoldX | 0.32 | 2.75 | 0.05 | 3.12 | 0.30 | 2.55 |

**Table S13. Performance comparison on multiple mutation blind test SM420 (non-redundant at multiple mutation level) forward mutations split by double and triple point mutations.**

|  | Overall | | Double | | Triple | |
| --- | --- | --- | --- | --- | --- | --- |
| Methods | Pearson | RMSE | Pearson | RMSE | Pearson | RMSE |
| **DDMut** | **0.66** | **1.83** | **0.65** | **1.94** | **0.72** | **1.42** |
| MAESTRO | 0.19 | 2.50 | 0.18 | 2.62 | 0.18 | 2.08 |
| FoldX | 0.32 | 2.75 | 0.26 | 2.86 | 0.52 | 2.34 |

**Table S14. Performance comparison on multiple mutation blind test SM444 (non-redundant at protein level) forward mutations split by stabilising and destabilising mutations.**

|  | Overall | | Stabilising | | Destabilising | |
| --- | --- | --- | --- | --- | --- | --- |
| Methods | Pearson | RMSE | Pearson | RMSE | Pearson | RMSE |
| **DDMut** | **0.49** | **2.45** | **0.39** | **2.46** | **0.35** | **2.47** |
| MAESTRO | 0.39 | 2.67 | 0.02 | 3.21 | 0.42 | 2.04 |
| FoldX | 0.41 | 2.76 | 0.22 | 2.90 | 0.35 | 2.63 |
| DDGun | 0.46 | 2.50 | 0.27 | 2.53 | 0.30 | 2.49 |

**Table S15. Performance comparison on multiple mutation blind test SM444 (non-redundant at protein level) forward mutations split by double and triple point mutations.**

|  | Overall | | Double | | Triple | |
| --- | --- | --- | --- | --- | --- | --- |
| Methods | Pearson | RMSE | Pearson | RMSE | Pearson | RMSE |
| **DDMut** | **0.49** | **2.45** | **0.50** | **2.37** | **0.45** | **2.82** |
| MAESTRO | 0.39 | 2.67 | 0.34 | 2.67 | 0.57 | 2.67 |
| FoldX | 0.41 | 2.75 | 0.34 | 2.80 | 0.65 | 2.51 |
| DDGun | 0.46 | 2.50 | 0.41 | 2.48 | 0.64 | 2.56 |


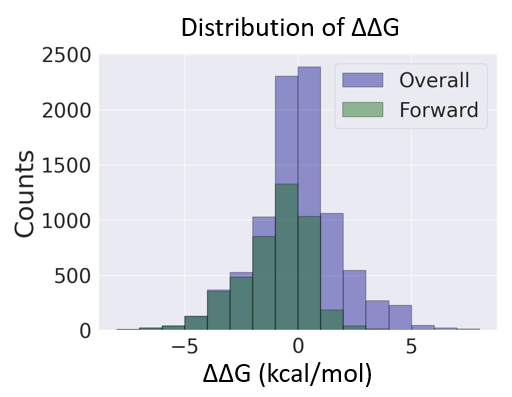


**Figure S1. ΔΔG distribution of training set S9028.** After including the hypothetical reverse mutations, ΔΔG follows a normal distribution with a mean value of 0 kcal/mol. This helps to balance the number of stabilising (ΔΔG ≥ 0) and destabilising (ΔΔG < 0) mutations.


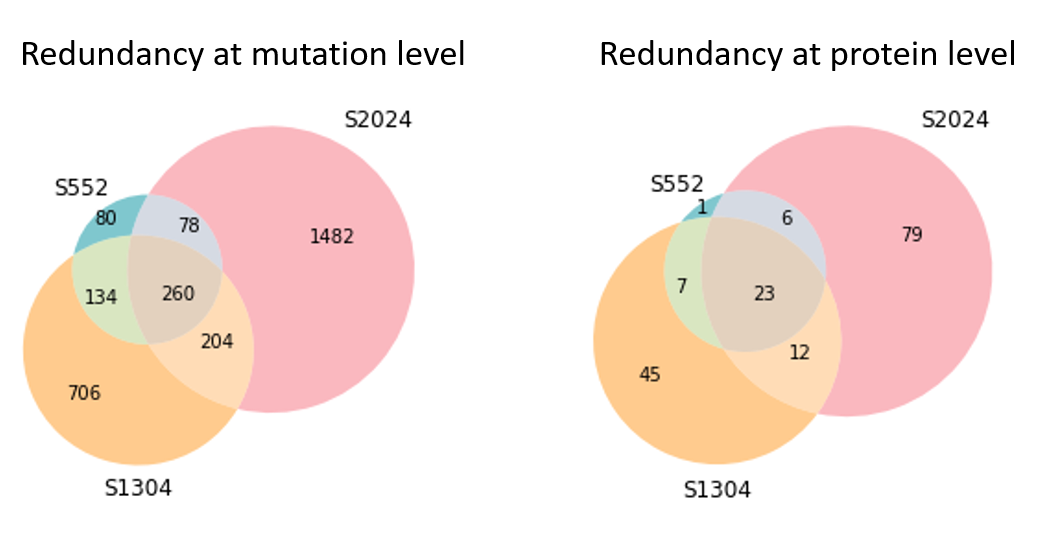


**Figure S2. Redundancy between the three blind test sets for single point mutations.** The left panel shows the overlaps between mutations in each dataset, and the right panel shows the overlaps between proteins in each dataset.


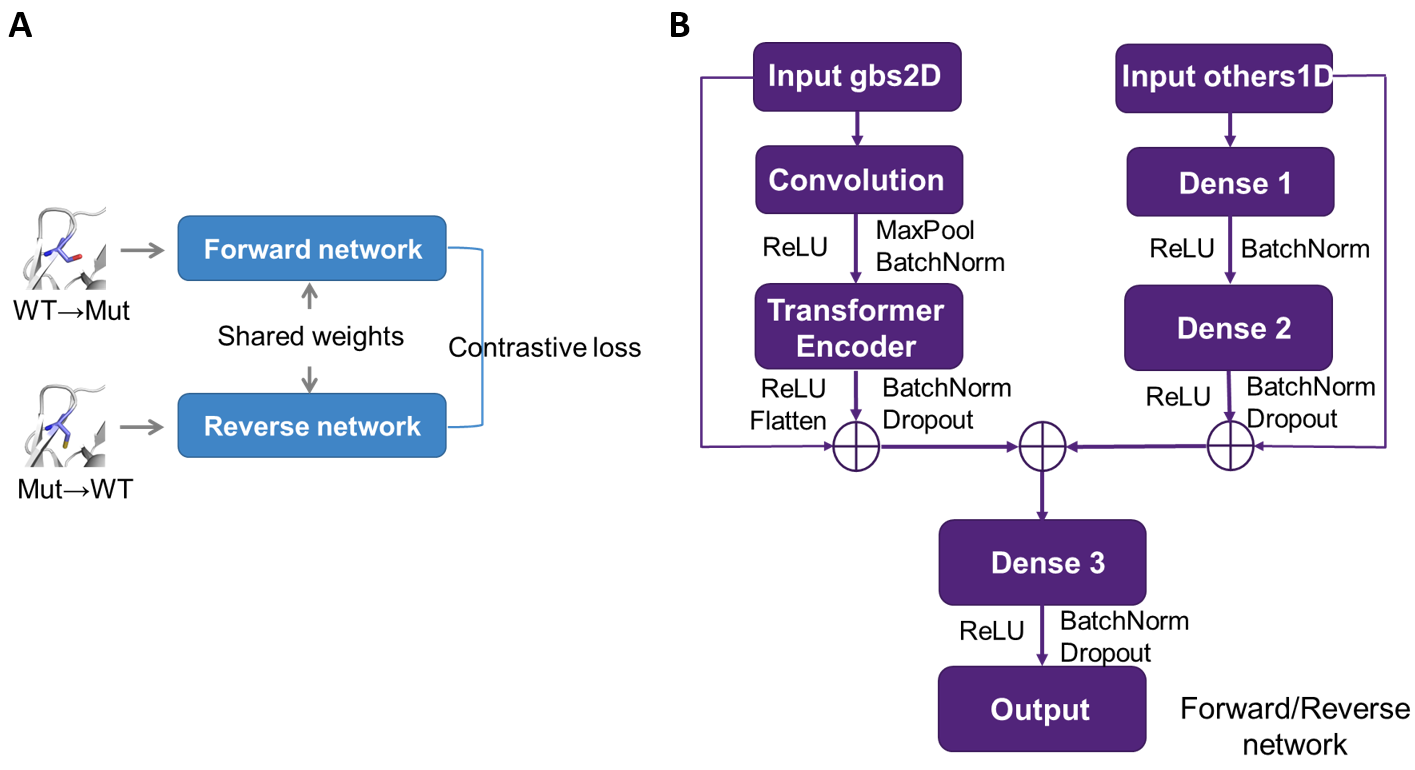


**Figure S3. DDMut Architecture.** (**A**) DDMut is a siamese network composed of one sub-network for forward mutations, and another one for reverse mutations. They share exactly the same architectures and weights. (**B**) The architecture for each sub-network.


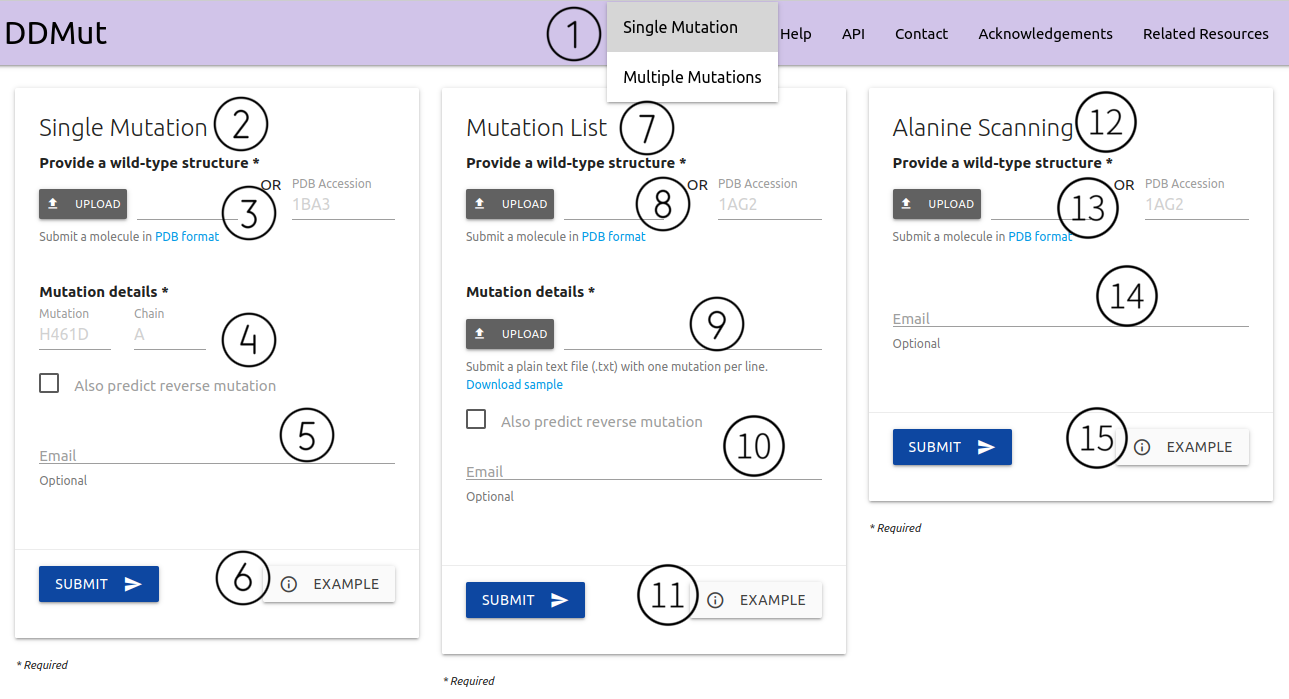


**Figure S4. DDMut input page for single point mutations.** The input page for single-point mutations can be accessed from the home page or via the top menu (1). Here, two options are available:

- Single Mutation (2) - Submitting one single point mutation
  - A structure must be provided as a file in PDB format or via PDB four letter accession code (3)
  - Mutation details are required to be provided in the form of *wild-type* code + *residue position* + *mutant* code (using the one letter amino acid code)(4). A chain identifier is also required. There is a checkbox to also predict reverse mutations, but it will take a bit longer to run.
  - If provided, an email will be sent to the user after the submission is processed (5)
  - Examples of inputs format and results are also available (6)
- Mutation List (7) - Submitting a list of single point mutations to be analysed and processed in batch
  - Like the Single Mutation option, here users are also required to input a structure must be provided as a file in PDB format or via PDB four letter accession code (8)
  - Mutation details are required to be provided as a plain text file (preferable format is .txt). Each mutation must be defined as *chain identifier* + *blank space* + *wild-type* code + *residue position* + *mutant* code (using the one letter amino acid code) (9). A sample file is available for download. There is a checkbox to also predict reverse mutations, but it will take a bit longer to run.
  - If provided, an email will be sent to the user after the submission is processed (10)
  - Examples of inputs format and results are also available (11)
- Alanine Scanning (12) - Submitting a structure and mutating every single residue to Alanine
  - A structure must be provided as a file in PDB format or via PDB four letter accession code (13)
  - If provided, an email will be sent to the user after the submission is processed (14)
  - Examples of inputs format and results are also available (15)


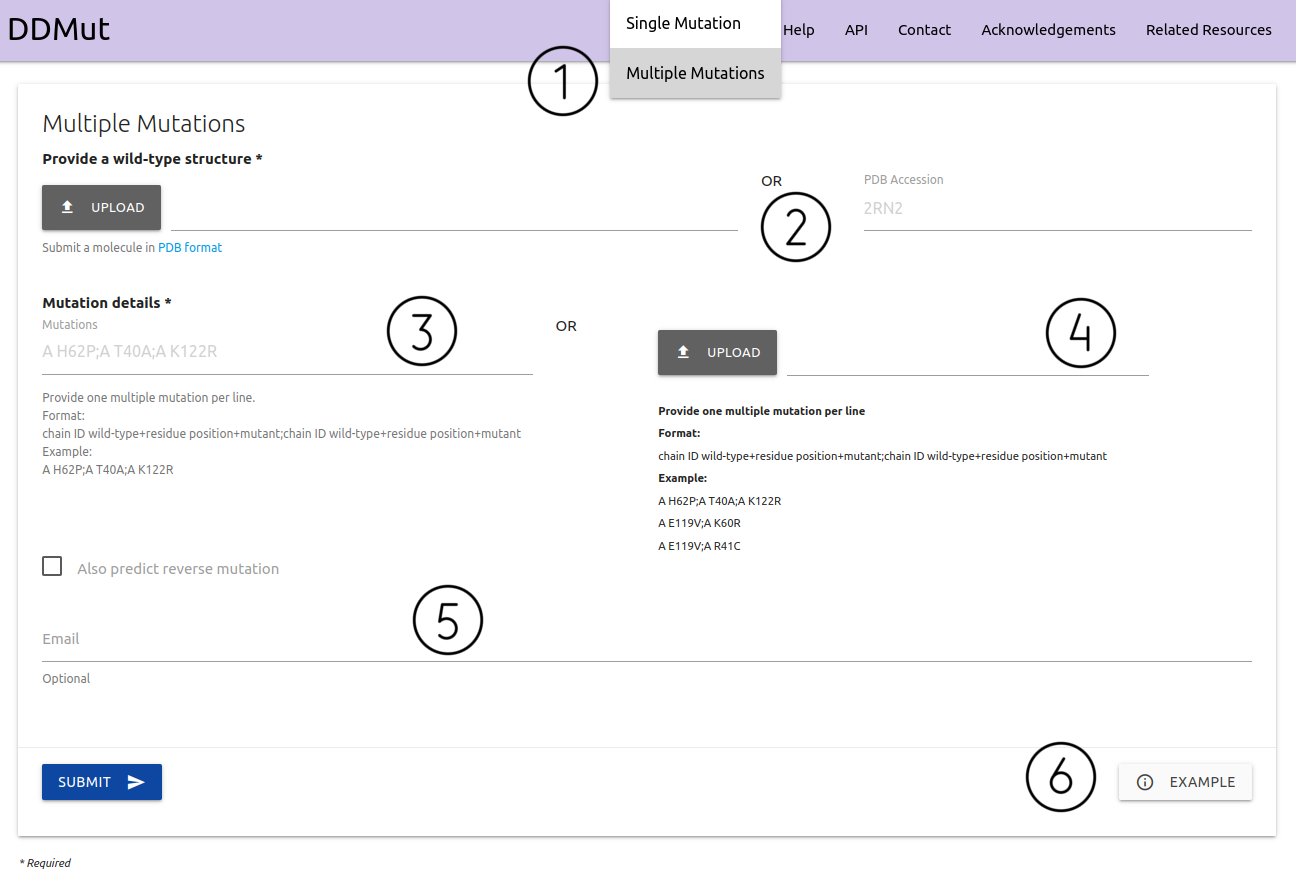


**Figure S5. DDMut input page for multiple point mutations.**The input page for running predictions for multiple mutations (double and triple mutants) is available from the home page or via the top menu (1)

- A structure must be provided as a file in PDB format or via PDB four letter accession code (2)
- Users can input a single entry (3) or a list of multiple mutations to be processed in batch (4). Entries are required to be provided in the form of Chain identifier + blank space + wild-type code + residue position + mutant code (using the one letter amino acid code) separated by a semi-colon (;). Example inputs are available. There is a checkbox to also predict reverse mutations
- If provided, an email will be sent to the user after the submission is processed (5)
- Examples of inputs format and results are also available (6)


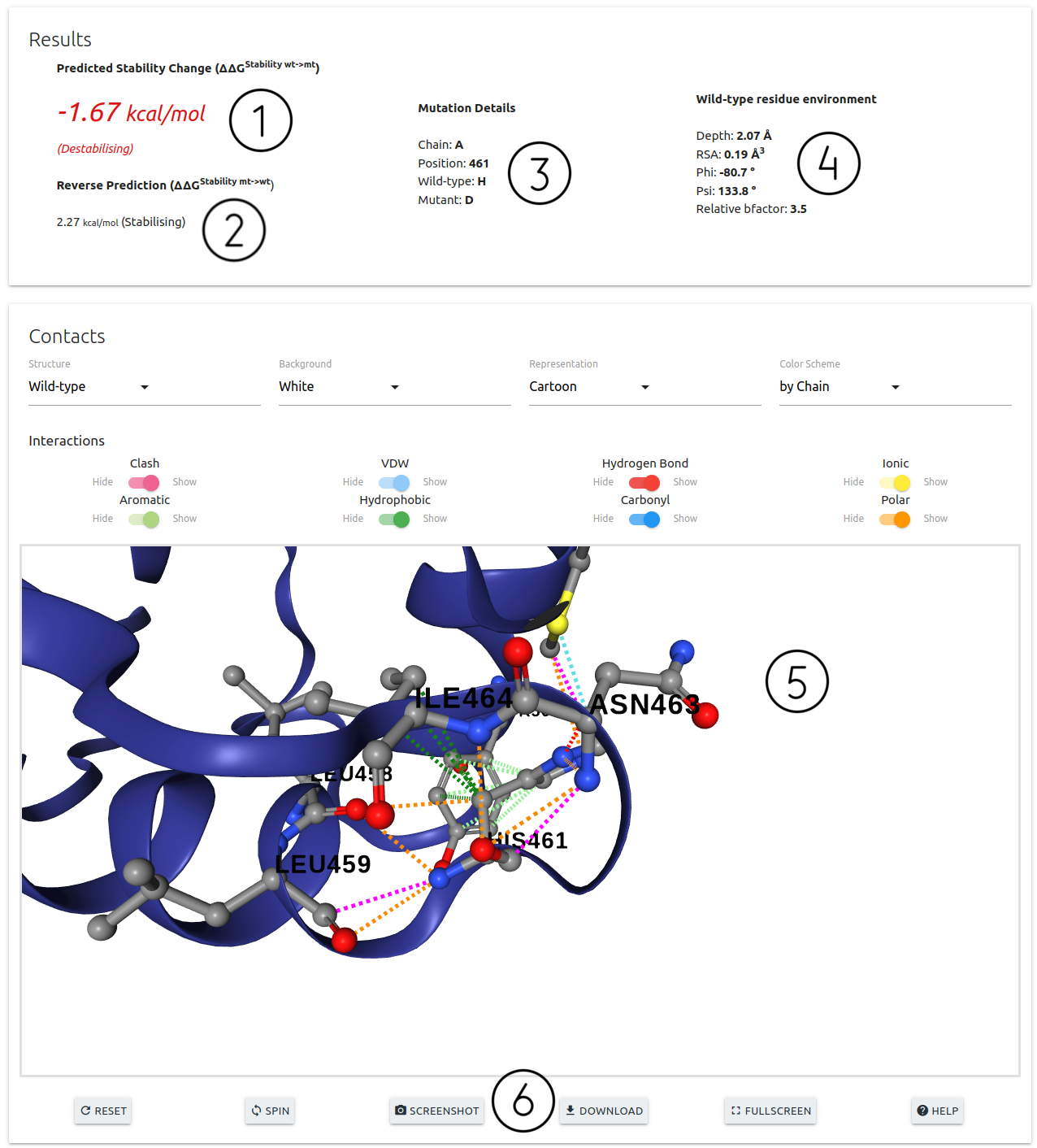


**Figure S6. The results page for Single Mutation.** On the results page for Single Mutation option:

- DDMut predictions for the forward (1) and reverse (2) mutations are shown on the top section of this page alongside with details on the input mutation (3) and wild-type residue environment (4)
- An interactive 3D viewer is available (5) allowing for the analysis of interatomic interactions for the wild-type residue. A set of controls are also available for customising the viewer
- Action buttons at the bottom of the viewer (6)


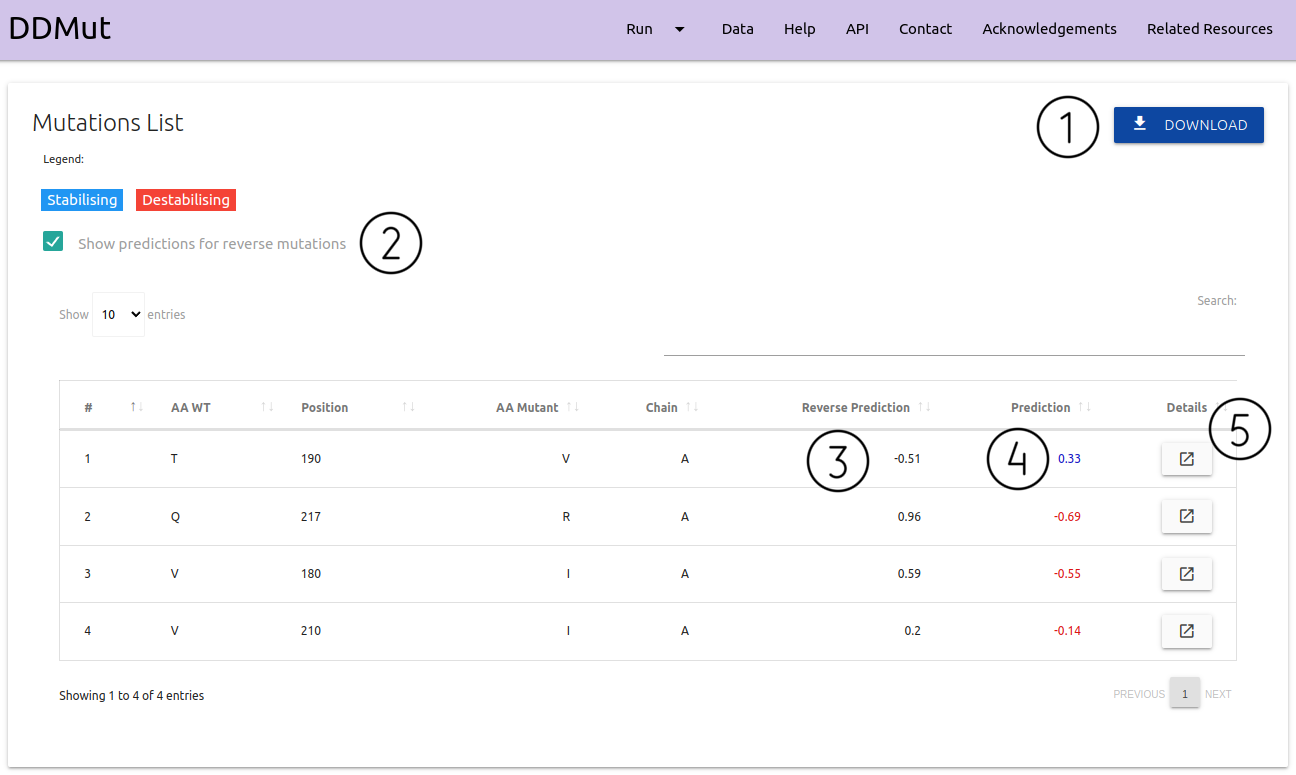


**Figure S7. The results page for Mutation List.** For Mutation List option:

- Results are summarised as a downloadable table (1)
- Details on the input mutations and predictions (4) for each entry are shown
- There is a checkbox (2) to show and hide the column for reverse predictions (3) if the user chose to also predict these
- Each entry can be visualised individually via the Details (5) button


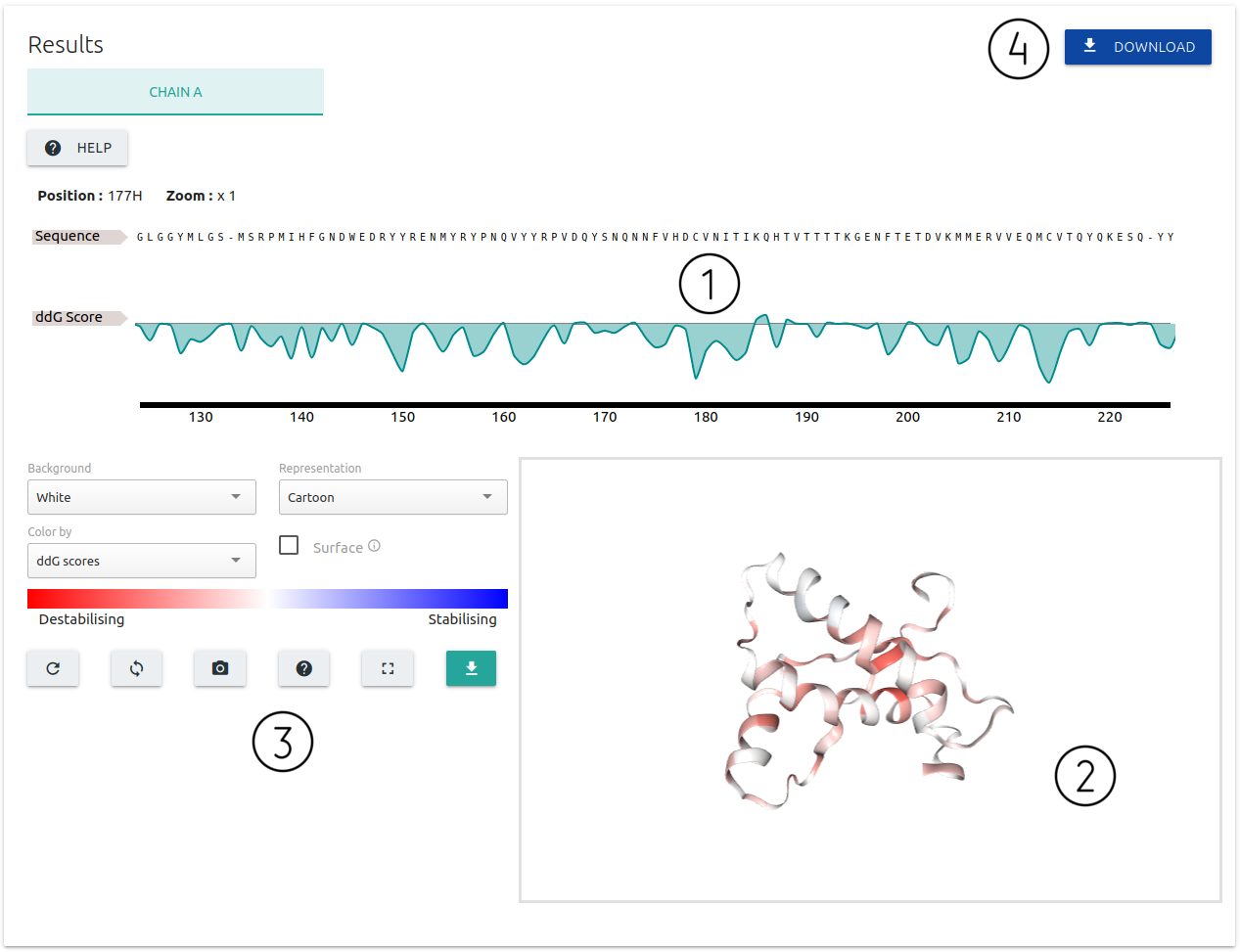


**Figure S8. The results page for Alanine Scanning.** For Alanine Scanning option:

- Results are summarised in relation to the sequence of amino acids present in each chain of the submitted protein structure (1), and also as an interactive 3D viewer (2) where scores for each residue are mapped onto the input structure
- A set of controllers and action buttons are available for customising the 3D viewer (3)
- There is a button to download the results as a table (4)

**
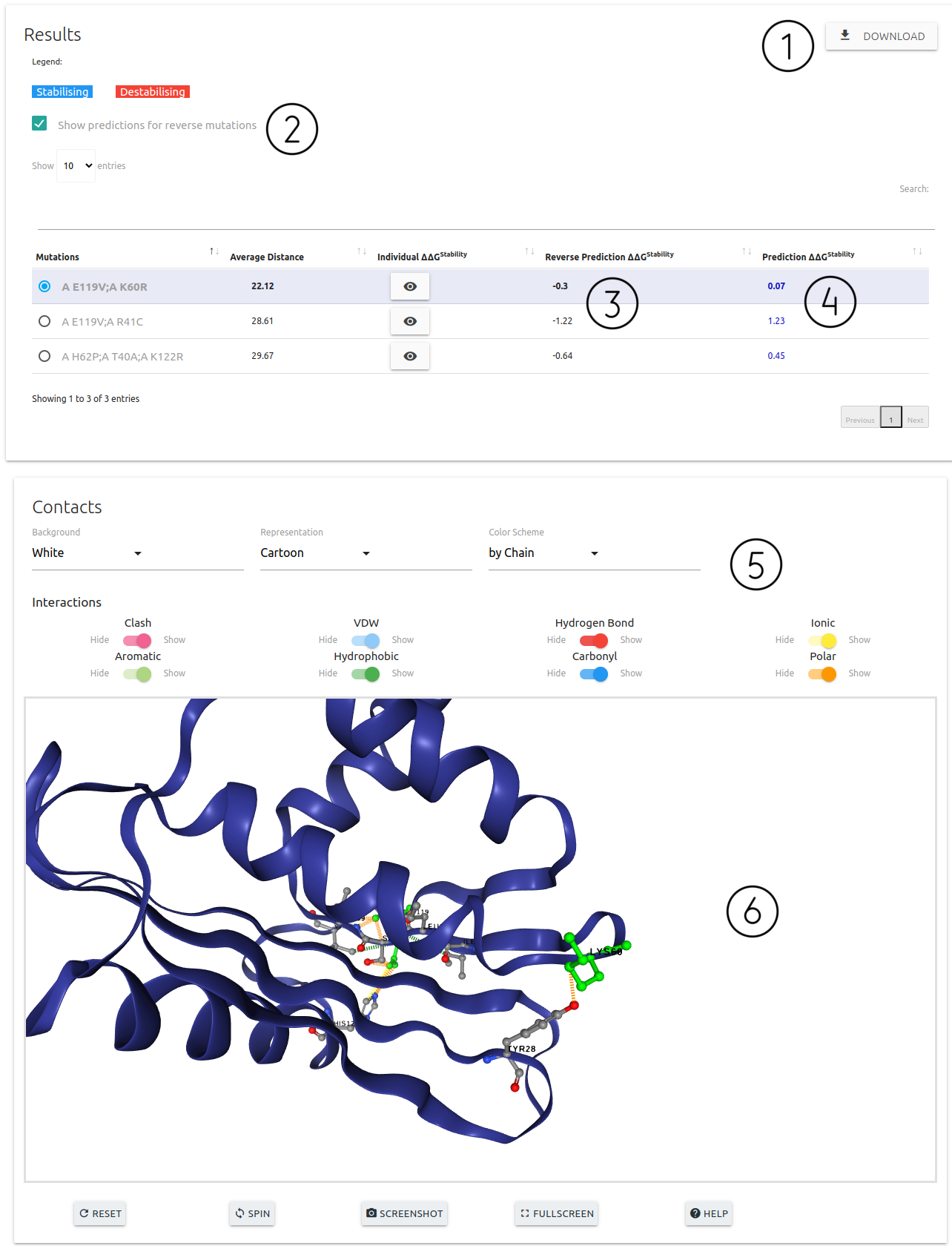
**

**Figure S9. The results page for Multiple Mutations.** Results are summarised as a downloadable table (1). Each entry on the table can be selected to be displayed on the 3D viewer (6)

- Details on the input mutations and predictions (4) for each entry are shown. Entries predicted as stabilising are shown in blue and destabilising in red. Individual ΔΔG_Stability_ for each point mutation can also be viewed by clicking the eye button. These are calculated using DDMut Single Mutation model.
- There is a checkbox (2) to show and hide the column for reverse predictions (3) if the user chose to also predict these
- A set of controls are available for customising the viewer (5)


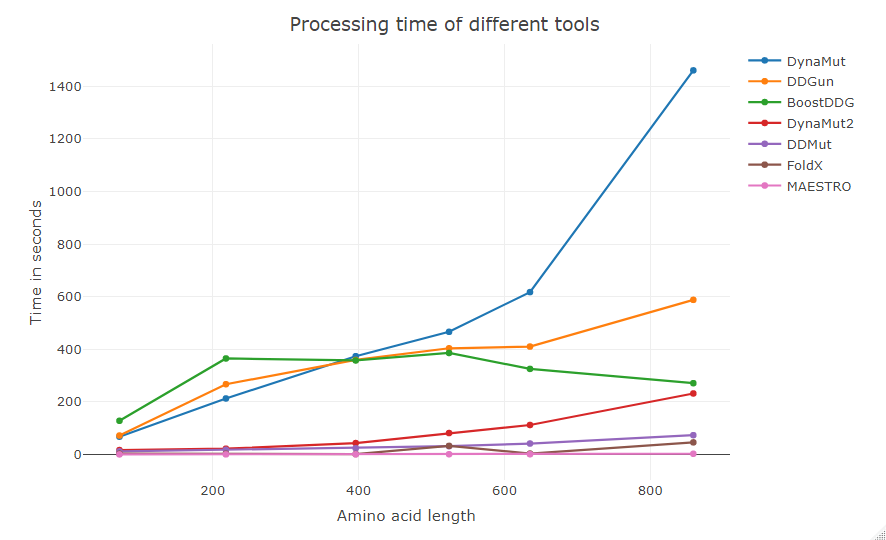


**Figure S10. Processing time of DDMut compared to other tools.** DDMut demonstrated competitive efficiency in predicting the effects of single point mutations across proteins of different sizes. The detailed time in seconds are shown in Table S2.


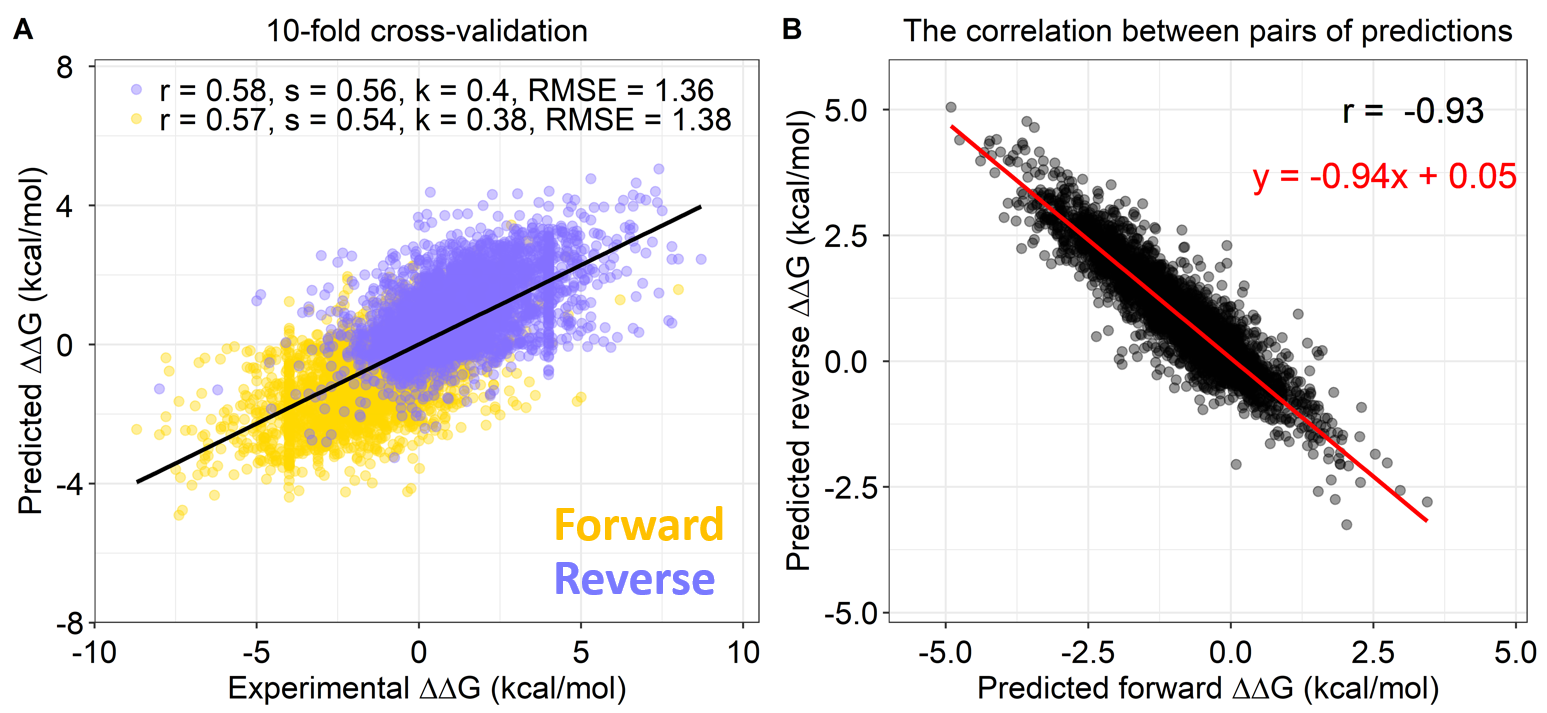


**Figure S11. DDMut cross-validation performance on forward and reverse mutations.** (**A**) DDMut achieved consistent performance on forward and reverse mutations. (**B**) The correlation between the forward and the hypothetical reverse mutations.


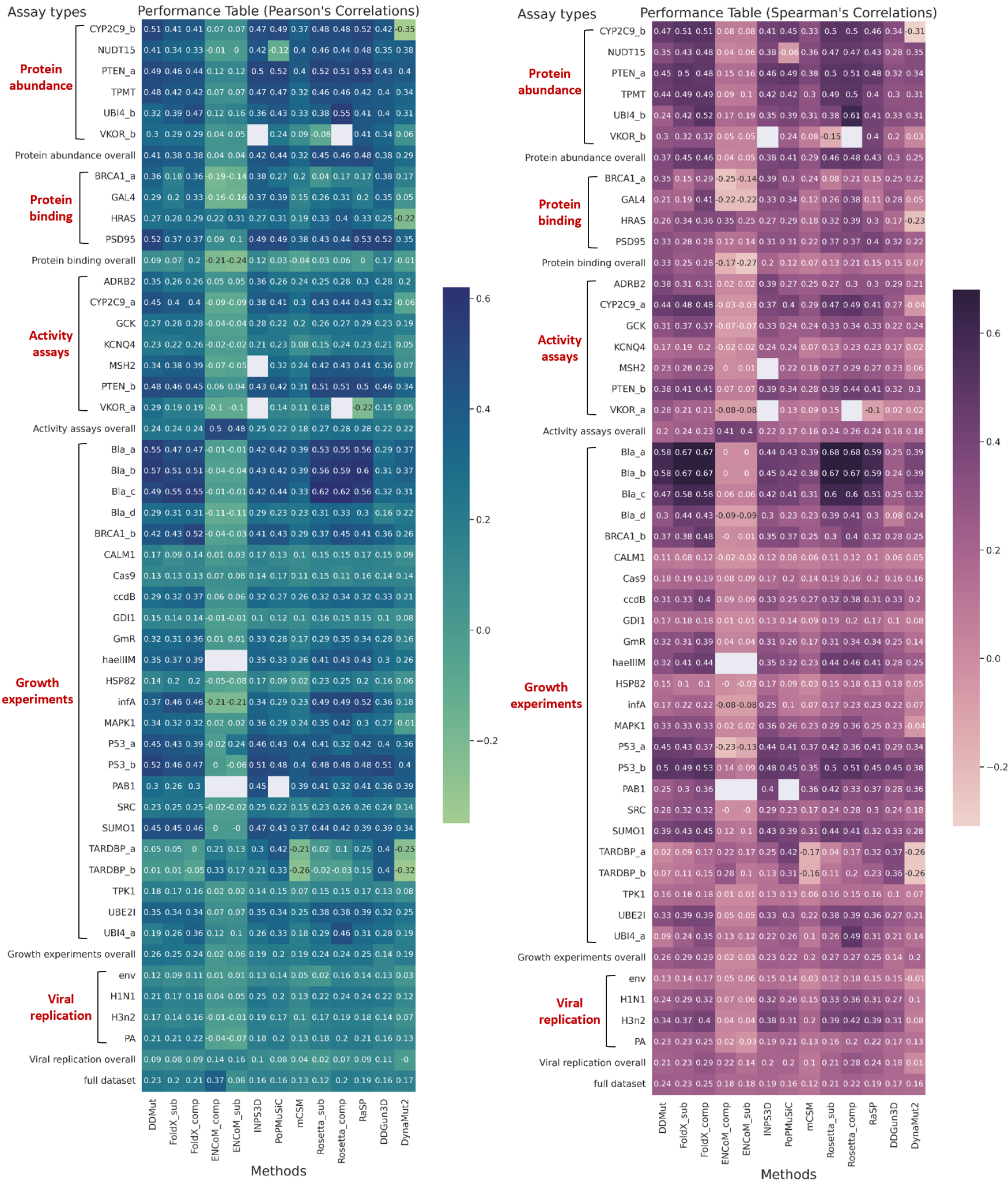


**Figure S12. Correlating protein stability and functional scores derived from deep mutational scans.** The performance of different protein stability predictors on DMS datasets with various assay types. The predictions by other tools were taken from Gerasimavicius *et al.* (4). The left panel shows Pearson's correlations, and the right panel shows Spearman’s correlations.

## **REFERENCES**

1. Kawashima, S. and Kanehisa, M. (2000) AAindex: amino acid index database. *Nucleic Acids Res*, **28**, 374. <http://dx.doi.org/10.1093/nar/28.1.374>

2. Cock, P.J., Antao, T., Chang, J.T., Chapman, B.A., Cox, C.J., Dalke, A., Friedberg, I., Hamelryck, T., Kauff, F., Wilczynski, B. *et al.* (2009) Biopython: freely available Python tools for computational molecular biology and bioinformatics. *Bioinformatics*, **25**, 1422-1423. <http://dx.doi.org/10.1093/bioinformatics/btp163>

3. Jubb, H.C., Higueruelo, A.P., Ochoa-Montano, B., Pitt, W.R., Ascher, D.B. and Blundell, T.L. (2017) Arpeggio: A Web Server for Calculating and Visualising Interatomic Interactions in Protein Structures. *J Mol Biol*, **429**, 365-371. <http://dx.doi.org/10.1016/j.jmb.2016.12.004>

4. Gerasimavicius, L., Livesey, B.J. and Marsh, J.A.J.b. (2023) Correspondence between functional scores from deep mutational scans and predicted effects on protein stability. 2023.2002. 2003.527007.
